# Reciprocal relations between prolonged grief and anger in homicidally bereaved people involved in a criminal trial: a four-wave cross-lagged panel model

**DOI:** 10.1017/S0033291725100809

**Published:** 2025-06-18

**Authors:** Lieke C.J. Nijborg, Maarten J.J. Kunst, Gerben J. Westerhof, Jos de Keijser, Lonneke I.M. Lenferink

**Affiliations:** 1 Department of Psychology, Health, and Technology, Faculty of Behavioral, Management, and Social Sciences, University of Twente, Enschede, the Netherlands; 2 Institute of Criminal Law and Criminology, Faculty of Law, Leiden University, Leiden, the Netherlands; 3 Department of Clinical Psychology and Experimental Psychopathology, Faculty of Behavioral and Social Sciences, University of Groningen, Groningen, the Netherlands; 4 Department of Clinical Psychology, Faculty of Social and Behavioral Sciences, Utrecht University, Utrecht, the Netherlands

**Keywords:** anger, bereavement, criminal justice system, grief, trauma

## Abstract

**Background:**

Anger may increase the risk for prolonged grief disorder (PGD) after violent loss. A source of anger for violently bereaved people can be the criminal proceedings that ensue following the loss. The present study explored the reciprocal associations between PGD and state anger and whether aspects of involvement in the criminal justice system (CJS) relate to PGD and state anger.

**Methods:**

We analyzed data of 237 MH17-bereaved people collected 67, 79, 88, and 103 months after the loss. Cross-lagged panel modeling was employed to examine the reciprocal associations between PGD and state anger. In the optimal model, we regressed PGD and state anger levels on different aspects of CJS involvement.

**Results:**

Higher PGD levels significantly predicted higher state anger levels at each wave (*β* = .112–.130) but not the other way around. This was found while constraining autoregressive and cross-lagged paths. When adding predictors and covariates to the model, PGD levels still consistently predicted state anger levels over time (*β* = .107–.121), with state anger levels predicting PGD levels to a lesser extent (*β* = .064–.070). None of the aspects of CJS involvement were related to either PGD or state anger levels.

**Conclusions:**

If replicated, a clinical implication could be that targeting PGD levels in treatment may reduce state anger levels and, to a lesser extent, vice versa. Also, CJS involvement does not seem to have an impact on PGD and state anger in people confronted with violent loss.

Around 3%–5% of the general population develops prolonged grief disorder (PGD) following natural bereavement (Rosner, Comtesse, Vogel, & Doering, 2021; Treml, Brähler, & Kersting, 2022; Treml, Linde, Brähler, & Kersting, 2024). Inherent to experiencing PGD is yearning, and/or preoccupation with, the deceased (American Psychiatric Association [APA], 2022). The Diagnostic and Statistical Manual of Mental Disorders, Fifth Edition, Text Revision (DSM-5-TR) states that PGD can be diagnosed when the person died at least one year earlier, and separation distress and accompanying symptoms are present for at least one month, resulting in functional impairment (APA, 2022). Note, from this point on, ‘PGD’ is used when disturbed grief reactions were assessed using the DSM-5-TR criteria, while ‘disturbed grief’ is used in cases when it concerns other conceptualizations, sometimes including the DSM-5-TR criteria. The risk of developing PGD after sudden loss, such as violent loss, is approximately four times higher than after natural loss (Doering, Barke, Vogel, Comtesse, & Rosner, 2022). Anger may increase the risk for disturbed grief after violent loss (Heeke, Kampisiou, Niemeyer, & Knaevelsrud, 2019).

Anger is useful for survival when one feels threatened (Novaco, 2010). Specifically, anger allows us to confront the threat head on by activating our fight response, thereby suppressing fear and allowing the person to re-establish a sense of control of the situation and promoting perseverance (American Psychological Association, 2022; Novaco, 2010). However, when feelings of anger remain after the threat is gone, anger may become pathological and hamper the grieving process. For instance, feelings of anger were found to be associated with more negative cognitions about the self after homicidal loss, which supposedly play a role in the maintenance of emotional distress (Boelen, van den Hout, & van den Bout, 2006; Boelen, van Denderen, & de Keijser, 2016). Also, frequently expressing and experiencing anger has been found to undermine social support that is necessary to cope with loss (Diong et al., 2005).

Feelings of anger are especially common after violent loss and often coincide with disturbed grief symptoms (Heeke et al., 2019; Rees, Tay, Savio, Maria Da Costa, & Silove, 2017). This may be partially explained by the loss being perceived as preventable. A cognitive-behavioral conceptualization of disturbed grief posits that negative cognitions, such as perceiving the loss as preventable, partially contribute to the development and maintenance of disturbed grief (Boelen et al., 2006). Specifically, if violently bereaved people believe the loss could have been prevented, they might direct their anger not only at the perpetrator but also themselves, thereby stimulating feelings of self-blame and guilt (Brent, Melhem, Donohoe, & Walker, 2009; Huh, Huh, Lee, & Chae, 2017; Kristensen, Heir, Herlofsen, Langsrud, & Weisæth, 2012; Mezey, Evans, & Hobdell, 2002). Indeed, directing anger at the self and feelings of self-blame and guilt are associated with higher disturbed grief levels (Field, Bonanno, Williams, & Horowitz, 2000; Lenferink, Nickerson, Kashyap, de Keijser, & Boelen, 2024; van Dijk, Boelen, de Keijser, Reitsma, & Lenferink, 2025; Wagner, Hofmann, & Grafiadeli, 2021). Thus, anger may play a more important role in the grief process following violent loss than natural loss, as the loss could have been prevented.

Several conceptualizations exist for anger. For instance, state anger refers to current feelings of anger that fluctuate over time (i.e., an emotional state), while trait anger refers to one’s proneness to experience anger over a longer time period (i.e., a personality trait) (Spielberger, Reheiser, & Sydeman, 1995). Cross-sectional research has shown that different conceptualizations of anger, such as state anger, are positively associated with disturbed grief levels (Anderson, 2009; Buiter et al., 2022; Lenferink, Nickerson, et al., 2024; Rees et al., 2017). Prior longitudinal research has examined reciprocal associations between different conceptualizations of anger and disorders that are commonly comorbid with disturbed grief, such as posttraumatic stress disorder (PTSD) (Lommen, Engelhard, van de Schoot, & van den Hout, 2014; Orth, Cahill, Foa, & Maercker, 2008) and depression (Galambos, Johnson, & Krahn, 2018; Spyropoulou & Giovazolias, 2022). For example, Orth et al. (2008) found that PTSD levels predicted state anger levels but not the other way around following (non)sexual assault. To the best of our knowledge, no study has examined the reciprocal associations between disturbed grief and anger. It seems worthwhile to examine this, as anger might compromise treatment outcomes (Foa, Riggs, Massie, & Yarczower, 1995; for an overview, see Lievaart, Franken, & Hovens, 2016; Rosen, Adler, & Tiet, 2013). This information can help determine whether feelings of anger need to be addressed before disturbed grief symptoms to optimize treatment outcomes.

A source of anger for violently bereaved people can be the criminal proceedings that ensue following the loss. In qualitative research, violently bereaved people have commonly expressed feelings of anger toward the criminal justice system (CJS) relating to, e.g., treatment by professionals, such as judges, prosecutors, and defense lawyers (Bertollini, 2006; Englebrecht, Mason, & Adams, 2014; Reed & Caraballo, 2022; Stretesky, Shelley, Hogan, & Unnithan, 2010), as well as the outcomes of the proceedings (Eagle, 2020; Malone, 2007). Moreover, if disturbed grief and anger levels are reciprocally related, then certain aspects of the CJS may be related to both disturbed grief and anger levels. For instance, a cross-sectional study showed that violently bereaved people who intended to follow court hearings or deliver a written or an oral statement reported higher disturbed grief levels, and these associations were partially or fully mediated by state anger levels (Buiter et al., 2022). Nevertheless, this study was cross-sectional and concerned intentions to participate. Thus, the research design makes it impossible to draw conclusions regarding the directionality of the relationship between disturbed grief and state anger, and whether actual participation in the CJS relates to both.

The present study explored the reciprocal associations between PGD and state anger using four annual measurement occasions. The reciprocal associations were examined in a sample of people bereaved by a plane disaster, the resulting deaths of which were ruled murders by the Netherlands Public Prosecution Service. Also, we explored whether aspects of CJS involvement, i.e., satisfaction with CJS professionals and the verdict, whether the person delivered a statement and received monetary compensation, and the number of statements the person listened to, relate to PGD and state anger.

## Methods

### Procedure and participants

The analytical design of the study was pre-registered on the Open Science Framework (ID = 35zmu). This cross-lagged analytic study is one of several studies evaluating the psychosocial outcomes of people bereaved by the MH17 plane disaster in 2014 (Buiter et al., 2022; Lenferink, de Keijser, Smid, Djelantik, & Boelen, 2017; Lenferink, Nickerson, de Keijser, Smid, & Boelen, 2019; Lenferink, Nickerson, de Keijser, Smid, & Boelen, 2020; Nijborg, Kunst, Westerhof, de Keijser, & Lenferink, 2024), which resulted in the deaths of 298 people (Dutch Safety Board, 2015). The Netherlands Public Prosecution Service prosecuted four suspects for these deaths. Three of the suspects were sentenced to life imprisonment, while one was acquitted (see Rechtbank Den Haag, 2022). Data for the present study were collected in four waves using surveys. The surveys were available in Dutch and English. The first wave (hereafter: pre-trial) took place between February 17, 2020 and March 9, 2020. These data were collected right before the start of the criminal trial on March 9, 2020. The second wave (hereafter: pre-statement) took place between February 24, 2021 and May 25, 2021. These data were collected before participants were able to deliver a statement about the crime during the court hearings, which happened between September 6, 2021 and November 8, 2021. The third wave (hereafter: post-statement) took place between November 22, 2021 and January 3, 2022, after participants had had the opportunity to deliver a statement. The fourth wave (hereafter: post-trial) took place between February 21, 2023 and June 1, 2023, between approximately 3–6 months after the verdict was issued. People were recruited at pre- and post-trial (for details, see Buiter et al. (2022) and Nijborg, Westerhof, Kunst, de Keijser, and Lenferink (2024), respectively).

One hundred ninety-nine people participated at pre-trial, 129 at pre-statement, 103 at post-statement, and 172 at post-trial. A total of 237 people participated in at least one wave and were therefore included in the analyses. Data from the pre-trial assessment were used in one study (Buiter et al., 2022), and data from the pre- and post-statement assessments in another study (Nijborg, Kunst, et al., 2024). Ethical approval was obtained from a local ethics committee (PSY-1920-S-0171). All participants provided written informed consent.

### Measures

#### Traumatic Grief Inventory Self-Report Plus (TGI-SR+)

PGD intensity was assessed according to the DSM-5-TR criteria, using the Traumatic Grief Inventory Self-Report Plus (TGI-SR+) (Lenferink, Eisma, Smid, de Keijser, & Boelen, 2022). In the instructions, we referred to the death of the participants’ loved one(s) due to the plane disaster. Also, it was indicated that when confronted with multiple losses, the participant should focus on the loss considered most stressful and/or most on their mind. The questionnaire consists of 22 items (e.g., ‘In the past month, I had intrusive thoughts or images related to the person who died’) that the participant rated from one (never) to five (always) (Lenferink et al., 2022). A total PGD score (range: 10–50) was computed at each wave by combining the scores on 10 items (i.e., one, three, six, nine, 10, 11, 18, 19, 21, and the highest score on items two and eight) representing DSM-5-TR PGD symptoms (Lenferink et al., 2022). A total score ≥33 indicates probable PGD. Notably, for the cross-lagged panel model, the total PGD scores were adjusted. To explain, the DSM-5-TR PGD symptom ‘intense emotional pain related to the death’ is assessed using item two (‘I experienced intense emotional pain, sadness, or pangs of grief’) and item eight (‘I felt bitterness or anger related to his/her death’). However, item eight shows content overlap with the state anger measure. Therefore, to prevent inflating the associations between total PGD and state anger scores, only the score indicated on item two was used as indicator of intense emotional pain when calculating the total PGD scores for the cross-lagged panel model. The psychometric properties of the questionnaire are acceptable (Kokou‐Kpolou et al., 2022; Lenferink et al., 2022, 2023; Lenferink, Johnsen, Kristensen, Lie, & Sveen, 2024). Cronbach’s alphas ranged from .92 to .93 across the waves, irrespective of whether answers on item eight were considered in the reliability analysis.

#### State–Trait Anger Expression Inventory 2 (STAXI-2)

State anger was assessed using the 15-item State Anger scale of the State–Trait Anger Expression Inventory 2 (STAXI-2) (Spielberger, Sydeman, Owen, Marsh, & Maruish, 1999). The items (e.g., ‘I am mad’) were rated from one (not at all) to four (very much so) (Spielberger et al., 1999), indicating to what extent the content corresponds to the participant’s emotional state at the time the survey was completed. A total state anger score (range: 15–60) was computed at each wave by summing all item scores. As there is no established cut-off score available for what level of state anger is considered to be above average, we compared the state anger mean at each wave to the mean of the general population (i.e., *M* = 18.72, *SD* = 7.08; Lievaart et al. (2016)). The psychometric properties of the STAXI-2 are acceptable (Dutch translation: Lievaart et al., 2016). Cronbach’s alphas ranged from .93 to .96 across the waves.

#### Satisfaction with treatment by CJS professionals

At post-trial, four items were utilized to evaluate the degree of satisfaction with the (1) the Netherlands Public Prosecution Service, (2) the criminal trial, (3) the judges, and (4) the defense team. Participants rated these items from one (very dissatisfied) to 10 (very satisfied). The item scores were summed (range: 4–40), representing the degree of satisfaction with treatment by CJS professionals. Cronbach’s alpha was .68 for the four items.

#### Satisfaction with verdict

At post-trial, one self-developed item was used to evaluate the degree of satisfaction with the verdict (‘How satisfied are you with the verdict?’). This item was rated from one (very dissatisfied) to 10 (very satisfied).

#### Statement delivery

At post-statement, one self-developed item was used to determine whether the participant delivered an oral or written statement (‘Did you yourself deliver an oral statement or did you draw up a written statement?’). Answer options were: 1 = yes, I submitted a written statement, 2 = yes, I delivered an oral statement, 3 = no, a loved one submitted a written statement, 4 = no, a loved one delivered an oral statement, 5 = no, nor I nor my loved ones submitted a written statement or delivered an oral statement. The answer options were dichotomized (i.e., 0 = no, 1 = yes). The group coded as no (0) comprises people who did not have the opportunity to deliver a statement and those that had the opportunity to deliver a statement but decided not to. To elaborate, Dutch criminal law states that relatives of the deceased have the right to deliver an oral statement, yet not all bereaved people were given the option (e.g., a maximum of three relatives per deceased person).

#### Receipt of monetary compensation

At post-trial, one self-developed item was used to determine whether the participants received monetary compensation for their loss (‘Did you receive compensation for your damages?’). Answer options were 0 = no, 1 = yes, I received compensation for the pain and suffering I experienced due to the loss of my loved one(s), 2 = yes, I received compensation for the material damages that I sustained as a result from the plane disaster, 3 = yes, I received compensation for both the pain and suffering I experienced and the material damages I sustained. Again, the answer options were dichotomized. The group coded as no (0) comprises people who could not apply for compensation, people who decided not to apply, and those who indicated that they applied for compensation but did not receive any.

#### Passive involvement

At post-statement, one self-developed item was used to evaluate the number of statements the participant had listened to (‘How many statements did you listen to, either while being physically present or online, concerning the MH17 disaster in court?’). Answer options were 1 = none, 2 = 1–10, 3 = 11–20, 4 = 21–40, 5 = 41–60, 6 = 61–80, 7 = 81, or more. The number of statements listened to was treated as a continuous variable and as a proxy measure for the extent each participant followed the criminal proceedings.

#### Background and loss-related characteristics

Based on prior research (Heeke et al., 2019; Kokou‐Kpolou et al., 2022), biological sex (1 = male, 2 = female), level of education (1 = primary education, 2 = middle school, 3 = secondary vocational education, 4 = university [of applied sciences]), and relationship to the deceased (1 = child, 2 = partner/spouse, 3 = sibling, 4 = parent, 5 = other) were assessed. Biological sex was recoded as 0 = male and 1 = female. Level of education was recoded as 0 = other than university (of applied sciences) and 1 = university (of applied sciences). As people often lost more than one loved one, closest relationship to the deceased was determined (range: child [closest], partner/spouse, parent, sibling, to other [most distant]). Then, relationship to the deceased was recoded as 0 = other than child or partner/spouse and 1 = child or partner/spouse.

#### Statistical analyses

The first aim of the study – examining the reciprocal associations between PGD (as defined in the DSM-5-TR) and state anger – was fulfilled by performing cross-lagged panel modeling in Mplus (Version 8; Muthén & Muthén, 1998–2017). The total PGD and state anger scores at each of the four waves were modeled, including cross-lagged and autoregressive paths. Cross-lagged paths were investigated to determine whether total PGD scores at one wave predict total state anger scores at the subsequent wave, and vice versa. Autoregressive paths were added to take into account total PGD and state anger scores reported at the previous wave. Specifically, total PGD scores in one wave were regressed on total PGD scores in the previous waves. Similarly, total state anger scores in one wave were regressed on total state anger scores in the previous waves. Based on prior research (Buiter et al., 2022), we expected, and therefore took into account, correlations between total PGD and state anger scores assessed at the same wave. Correlations were either interpreted as being weak (.10 ≤ *ρ* < .30), moderate (.30 ≤ *ρ* < .50), or strong (*ρ* ≥ .50) (Cohen, 1988).

The optimal model was determined by adding constraints (i.e., assuming equal associations) in a stepwise manner (i.e., model trimming; Kline, 2016). In the first step, the fit of an unconstrained model was tested. In the second step, the autoregressive paths between the total PGD scores were constrained to be equal across all waves, and the autoregressive paths between the total state anger scores were constrained to be equal across all waves. In the third step, cross-lagged paths were constrained in addition to the autoregressive paths described in step two. Specifically, the cross-lagged paths between the total PGD scores at each wave and total state anger scores in the subsequent wave were constrained to be equal across all waves. Additionally, the cross-lagged paths between the total state anger scores at each wave and the total PGD scores in the subsequent wave were constrained to be equal across all waves. In the fourth and final step, in addition to constraining the aforementioned autoregressive and cross-lagged paths, the associations between the total PGD and state anger scores were constrained to be equal across all waves when assessed concurrently. Notably, the models were first estimated without random intercepts. In a second round of analyses, random intercepts were added (i.e., separating between- and within-person effects). As non-convergence occurred, the cross-lagged panel models without random intercepts were reported.

Four fit indices were used to select the optimal model. The Comparative Fit Index (CFI) and the Tucker-Lewis Index (TLI) (>.90 equals acceptable fit; >.95 equals good fit), as well as the Root Mean Square Error Approximation (<.10 equals acceptable fit; <.06 equals good fit) and Standardized Root Mean Square Residual (<.08 equals acceptable fit; <.06 equals good fit) (Baribeau et al., 2022). The most parsimonious model was preferred, i.e., the model with the least structural paths that fits the data well (Little, 2013). The effect sizes of the cross-lagged paths were interpreted using small (.03), medium (.07), and large (.12) as surrounding anchors (Orth et al., 2024).

Regarding missing data, the total PGD score of one participant (<1%) was missing at pre-trial. Moreover, the total state anger scores of eight participants (4%) were missing at pre-trial, of two participants (2%) at post-statement, and also of two participants (1%) at post-trial. Missing data on the total PGD and state anger scores were accounted for using full information maximum likelihood.

The second aim of the study – examining whether aspects of CJS involvement relate to PGD and state anger over time – was fulfilled by regressing the total PGD and state anger scores on each aspect of CJS involvement in the optimal model. To specify, the total PGD and state anger scores at post-trial were regressed on satisfaction with treatment by CJS professionals, satisfaction with verdict, and receipt of monetary compensation. Additionally, the total PGD and state anger scores at post-statement and post-trial were regressed on statement delivery and passive involvement. Biological sex, level of education, and relationship to the deceased were added to the analyses as covariates by regressing the total PGD and state anger scores at pre-trial on these covariates. Two-tailed tests were used with *p* < .05 indicating a significant association.

For missing data on the aspects of CJS involvement and covariates, multiple imputation was used. Specifically, 100 datasets were generated. Based on prior research (Nijborg, Westerhof, et al., 2024), there seems to be no reason for concern regarding multicollinearity between the predictors (i.e., aspects of CJS involvement) and covariates (i.e., background and loss-related characteristics).

## Results

### Sample characteristics

The majority of the sample was female, born in the Netherlands, university-educated, lost multiple loved ones in the MH17 plane disaster, and most often lost (at least) a sibling (see Table 1). Concerning probable PGD caseness, 54 participants (27%) scored above the cut-off score at pre-trial, 26 (20%) at pre-statement, 15 (15%) at post-statement, and 20 (12%) at post-trial. On average, the state anger scores were significantly higher than those found in the general population at pre-trial (*t* (1400) = 5.09, *p* < .001, *d* = .40) and pre-statement (*t* (1338) = 2.94, *p* = .003, *d* = .27), but not at post-statement (*t* (1310) = 1.26, *p* = .208, *d* = .13) and post-trial (*t* (1379) = 1.84, *p* = .07, *d* = 0.15). Seventy-nine participants (33%) participated in one wave, 37 (16%) in two waves, 34 (14%) in three waves, and 87 (37%) in all four waves.

**Table 1.** Sample characteristics (N = 237)

Background and loss-related characteristics, and aspects of CJS involvement	
Biological sex, *N* (%)	
Male	88 (37)
Female	149 (63)
Country of birth, *N* (%)	
The Netherlands	186 (79)
Malaysia	16 (7)
Australia	11 (5)
Belgium	7 (3)
The United Kingdom of Great Britain and Northern Ireland	6 (3)
Indonesia	3 (1)
Germany	2 (1)
Other	6 (3)
Age at the time of the disaster, *M* (*SD*), range	47.66 (15.91), 14–86
Level of education, *N* (%)	
University (of applied sciences)	162 (68)
Secondary vocational education	31 (13)
Secondary school	43 (18)
Primary school	1 (<1)
Number of losses, *N* (%)	
One	93 (39)
Two	74 (31)
Three	30 (13)
Four	36 (15)
Five	3 (1)
Six	1 (<1)
Deceased relative is my…^a^, *N* (%)	
Child	58 (25)
Partner/spouse	9 (4)
Parent	30 (13)
Sibling	77 (33)
Other	62 (26)
Time since loss(es) in months, *M* (*SD*), range	
Pre-trial	67.01 (0.07), 67–68
Pre-statement	79.27 (0.66), 79–82
Post-statement	88.05 (0.22), 88–89
Post-trial	103.35 (0.69), 103–106
Satisfaction with treatment by CJS professionals, *M* (*SD*), range	31.92 (5.38), 19–40
Satisfaction with verdict, *M* (*SD*), range	8.04 (2.04), 1–10
Statement delivery, *N* (%)	
Oral statement, themselves	24 (28)
Written statement, themselves	13 (15)
Oral statement, someone else	15 (17)
Written statement, someone else	5 (6)
Neither themselves nor someone else	29 (34)
Receipt of monetary compensation, *N* (%)	
Immaterial compensation	56 (33)
Material compensation	3 (2)
Both	4 (2)
None	106 (63)
Number of statements listened to, *N* (%)	
None	29 (29)
1–10	43 (43)
11–20	8 (8)
21–40	8 (8)
41–60	3 (3)
61–80	2 (2)
81 or more	8 (8)

*Note.* Some characteristics had missing values.

Abbreviation: CJS = Criminal Justice System.

a When the participant lost multiple loved ones, only the participant’s closest relationship was used in the analyses ordered from child, partner/spouse, parent, sibling to other.

### Preliminary analyses

Means, standard deviations, ranges of, and correlations between the total scores of PGD and state anger at all waves are displayed in Table 2. PGD levels were significantly, positively, and strongly correlated across all waves. The same applies to state anger levels. PGD and state anger levels were significantly, moderately to strongly positively correlated across all waves.

**Table 2.** Descriptives and bivariate associations between PGD and state anger across four waves (N = 237)

Construct	Descriptive statistics				PGD			State anger			
	*N*	*M*	*SD*	Observed range	Pre-statement	Post-statement	Post-trial	Pre-trial	Pre-statement	Post-statement	Post-trial
PGD (range: 10–50)											
Pre-trial	198	26.82	9.54	10–50	.764	.696	.763	.582	.398	.394	.465
Pre-statement	129	24.43	8.87	10–46		.864	.756	.541	.496	.548	.445
Post-statement	103	23.79	8.41	10–45			.798	.452	.436	.472	.469
Post-trial	172	23.51	8.16	10–47				.553	.538	.516	.573
State anger (range: 15–60)											
Pre-trial	191	21.63	8.84	15–59					.681	.632	.600
Pre-statement	129	20.71	9.11	15–60						.694	.585
Post-statement	101	19.65	7.68	15–58							.733
Post-trial	170	19.78	6.80	15–44							

*Note.* Due to non-normality of total scores, Spearman’s rho correlations are reported.

Abbreviation: PGD = Prolonged Grief Disorder.

All correlations are significant at *p* < .001.

### Temporal associations between PGD and state anger

The model with constrained autoregressive and cross-lagged paths (i.e., model 3) was the only model that had acceptable CFI and TLI estimates (see Supplementary Table 1). Therefore, it was decided that the model with constrained autoregressive and cross-lagged paths is the optimal model.

**Figure 1.**
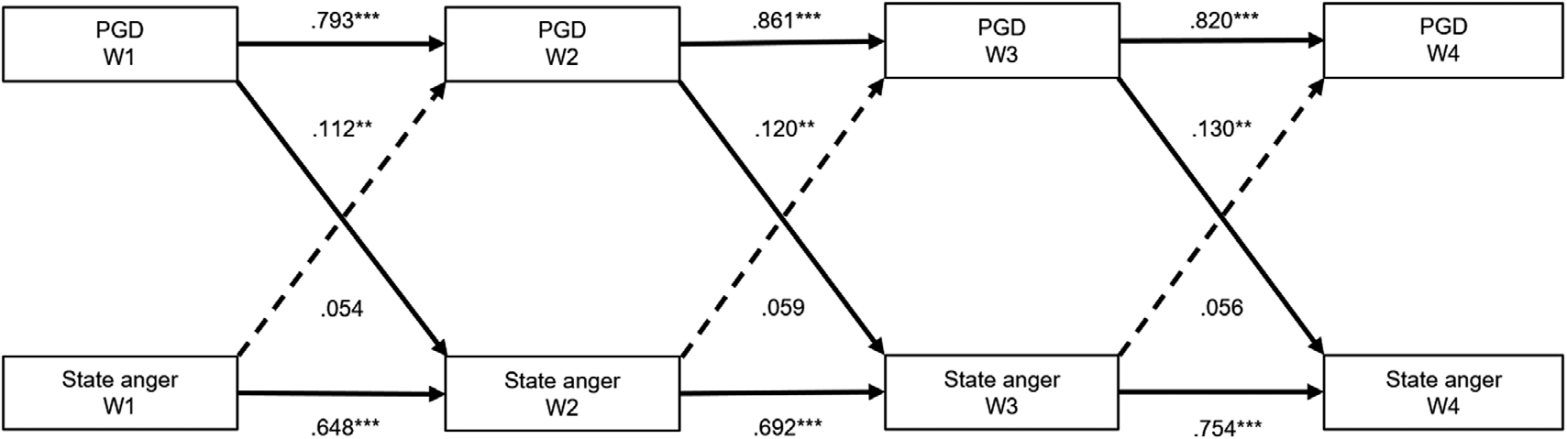
Standardized autoregressive and cross-lagged paths between PGD and state anger (N = 237). *Note.* *** *p* < .001, ** *p* < .01. Abbreviations: PGD = Prolonged Grief Disorder; W1 = pre-trial (67 months post-loss); W2 = pre-statement (79 months post-loss); W3 = post-statement (88 months post-loss); W4 = post-trial (103 months post-loss). The concurrent associations between PGD and state anger at each wave are not shown. Dashed lines represent non-significant paths.

PGD levels were found to be a consistent longitudinal predictor of state anger levels (and not vice versa). Specifically, higher PGD levels significantly predicted higher state anger levels at each wave, while state anger levels did not predict subsequent PGD levels. The effect sizes of these cross-lagged paths were medium to large or large. The standardized estimates of the optimal cross-lagged panel model are shown in Figure 1 and the unstandardized estimates in Supplementary Figure 1. When adding predictors and covariates to the model, PGD levels still consistently predicted state anger levels over time (*β* = .107–.121), with state anger levels predicting PGD levels to a lesser extent (*β* = .064–.070). The standardized estimates are shown in Supplementary Figure 2 and the unstandardized estimates in Supplementary Figure 3.

### Associations between aspects of CJS involvement and PGD and state anger

None of the aspects of CJS involvement were related to PGD or state anger levels at post-statement or post-trial (see Table 3). Regarding the covariates, university-educated people reported significantly lower PGD and state anger levels at pre-trial than not university-educated people. Moreover, people who lost (at least) a child or partner/spouse reported significantly higher PGD levels at pre-trial than people who lost other loved ones. Biological sex was unrelated to PGD and state anger levels at pre-trial.

**Table 3.** Predictors and covariates of PGD and state anger (N = 237)

	Pre-trial			Post-statement			Post-trial		
	β	95% CI	SE	β	95% CI	SE	β	95% CI	SE
PGD									
Satisfaction with treatment by CJS professionals							0.02	[−0.11, 0.14]	0.06
Satisfaction with verdict							−0.11	[−0.25, 0.02]	0.07
Statement delivery				0.02	[−0.08, 0.11]	0.05	0.04	[−0.07, 0.15]	0.06
Receipt of monetary compensation							0.09	[0.00, 0.19]	0.05
Passive involvement				0.07	[−0.02, 0.15]	0.04	−0.01	[−0.12, 0.10]	0.05
Biological sex	0.09	[−0.04, 0.22]	0.07						
Level of education	−0.22**	[−0.35, −0.09]	0.07						
Relationship to the deceased	0.20**	[0.07, 0.33]	0.06						
State anger									
Satisfaction with treatment by CJS professionals							−0.02	[−0.16, 0.12]	0.07
Satisfaction with verdict							−0.04	[−0.18, 0.11]	0.07
Statement delivery				−0.08	[−0.21, 0.05]	0.07	0.00	[−0.12, 0.12]	0.06
Receipt of monetary compensation							0.05	[−0.05, 0.15]	0.05
Passive involvement				0.12	[0.00, 0.24]	0.06	−0.01	[−0.13, 0.10]	0.06
Biological sex	0.01	[−0.12, 0.15]	0.07						
Level of education	−0.19**	[−0.33, −0.05]	0.07						
Relationship to the deceased	−0.03	[−0.16, 0.11]	0.07						

*Note.* **p < .01.

Abbreviations: PGD = Prolonged Grief Disorder; CJS = Criminal Justice System; CI = Confidence Interval.

## Discussion

Our first aim concerned the examination of the reciprocal associations between PGD and state anger in 237 people who lost loved ones in the MH17 plane disaster. We analyzed four waves of data using cross-lagged panel modeling. These data were collected before, during, and after the criminal trial took place (i.e., between 5 and 9 years following the loss). Data collection was timed as such to fulfill our second aim, i.e., examining whether aspects of CJS involvement relate to PGD and state anger levels.

With respect to the first aim, without including predictors and covariates, PGD levels consistently predicted state anger levels over time but not vice versa. This aligns with a prior study that found that higher PTSD levels predicted higher state anger levels following (non)sexual assault but not vice versa (Orth et al., 2008). When adding predictors and covariates to the model, PGD levels still consistently predicted state anger levels over time, with state anger levels predicting PGD levels to a lesser extent. These findings concur with prior research suggesting that anger may contribute to the maintenance of emotional distress (Boelen et al., 2016; Diong et al., 2005; Lenferink, Nickerson, et al., 2024). Based on the findings, we are the first to suggest that feelings of anger after violent loss may result from disturbed grief, and that state anger levels may to some extent maintain disturbed grief levels and therefore hinder recovery. Yet, the absence of prior research and the relatively small sample size prevent us from drawing firm conclusions. Future studies may want to examine whether the found associations generalize to other populations, as well as different conceptualization of disturbed grief and anger. If replicated, a clinical implication could be that targeting PGD levels in treatment may reduce state anger levels and to a lesser extent vice versa.

With respect to our second aim, none of the aspects of CJS involvement were related to PGD or state anger. This contrasts the belief of policy makers, who suggest that CJS involvement can promote one’s ‘emotional recovery’ from the crime (Kunst, Popelier, & Varekamp, 2015). The results underscore the importance of examining whether aspects of (actual) CJS involvement relate to PGD and state anger levels using a longitudinal design to ensure the robustness of the findings (cf. Buiter et al., 2022). Furthermore, being higher educated was negatively related to PGD and state anger levels, and being closer related to the deceased was positively related to PGD levels. Thus, background and loss-related characteristics seem to be better predictors of PGD and state anger levels than CJS involvement following violent bereavement.

The strengths of the present study are twofold. First, we examined the reciprocal associations between PGD and state anger using four waves of data. Due to this, we were able to provide a detailed picture of the reciprocal relationships between disturbed grief and state anger and draw tentative conclusions regarding their temporal stability. Second, all participants lost (at least) one loved one due to the same cause. Thus, details regarding the loss and how the criminal justice system handled the case were identical for the entire sample, making the sample homogeneous.

Despite its strengths, the study has several limitations. As the waves were at least 9 months apart, subtle, short-term changes between PGD and state anger levels may not have been captured. Also, caution is warranted when generalizing our findings to violently bereaved people for whom the criminal proceedings took place during the first years following bereavement. Moreover, the homogeneity of the sample decreases the generalizability of the findings to other violently bereaved people. Additionally, adding a random intercept to the cross-lagged panel models resulted in non-convergence. Consequently, we were unable to separate between-person from within-person effects. Yet, associations on the within-person level may differ in strength, or even direction, from the between-person level (Hamaker, Kuiper, & Grasman, 2015; Nelemans et al., 2020). Furthermore, we examined PGD levels in a trait-like manner (i.e., retrospectively) and anger as a momentary state, similar to Orth et al. (2008). While this could have led to artificial directionality, this is likely negated by the strong correlations found over time for PGD and state anger separately, indicating relative temporal stability. Even so, future studies may want to examine the reciprocal associations between momentary PGD and state anger levels. Lastly, some participants reported having delivered a statement or received compensation while this was not legally possible, pointing toward self-report bias. Yet, this concerns around 1% of the sample. Similarly, multiple imputation does not account for certain participants not being legally allowed to deliver a statement or request compensation due to their relationship to the deceased. However, this concerns around 10% of the sample. Therefore, it is unlikely that this has affected our results in a meaningful way. Pending replication of our findings, the findings should be interpreted with caution.

To conclude, we are the first to examine the reciprocal associations between PGD and state anger. Using four waves of data collected in the context of a criminal trial, we found support for a bidirectional effect between PGD and state anger over time in a sample of violently bereaved people, with PGD predicting state anger to a greater extent than vice versa. Thus, reducing PGD levels to reduce state anger levels may be more effective than vice versa. Furthermore, aspects of CJS involvement did not predict PGD and state anger levels above and beyond previous levels, as well as background and loss-related characteristics. In short, the present study provides preliminary support for healthcare professionals targeting PGD levels in treatment having the potential to reduce state anger levels and, to a lesser extent, vice versa, and that CJS involvement does not seem to impact PGD and state anger levels in people confronted with violent loss.

## Supporting information

Nijborg et al. supplementary material Nijborg et al. supplementary material

## Data Availability

The pseudonymized datasets, data dictionary, and Mplus output files can be found on the Data Archiving and Networked Services (DANS) repository see: https://doi.org/10.17026/SS/YWBDAA.

## Supplementary material

The supplementary material for this article can be found at http://doi.org/10.1017/S0033291725100809.
