# Supplementary material for: Reciprocal relations between prolonged grief and anger in homicidally bereaved people involved in a criminal trial: a four-wave cross-lagged panel model
Source: Psychol Med. 2025 Jun 18;55:e169. doi: 10.1017/S0033291725100809 (PMC13120860; doi:10.1017/S0033291725100809)
Supplement: Nijborg et al. supplementary material [file S0033291725100809sup001.docx]

**Supplementary Table 1**

*Fit Indices for Cross-Lagged Panel Models for PGD and State Anger (N = 237)*

|  | **df** | **CFI** | **TLI** | **RMSEA [90% CI]** | **SRMR** |
| --- | --- | --- | --- | --- | --- |
| Model 1 | 12 | 0.946 | 0.878 | 0.126 [0.094, 0.159] | 0.056 |
| Model 2 | 16 | 0.925 | 0.873 | 0.128 [0.100, 0.157] | 0.092 |
| **Model 3** | **20** | **0.927** | **0.902** | **0.113 [0.087, 0.139]** | **0.097** |
| Model 4 | 23 | 0.859 | 0.834 | 0.146 [0.123, 0.170] | 0.194 |

*Note.* Model 1 is the unconstrained model, in Model 2 the autoregressive effects are constrained, in Model 3 the autoregressive and cross-lagged effects are constrained. In Model 4 the autoregressive effects, cross-lagged effects and effects between PGD and state anger at the same wave are constrained. The optimal model is in bold. PGD = Prolonged Grief Disorder; CFI = Comparative Fit Index; TLI = Tucker-Lewis Index; RMSEA = Root Mean Square Error of Approximation; CI = Confidence Interval; SRMR = Standardized Root Mean Square Residual.

**Supplementary Figure 1**

*
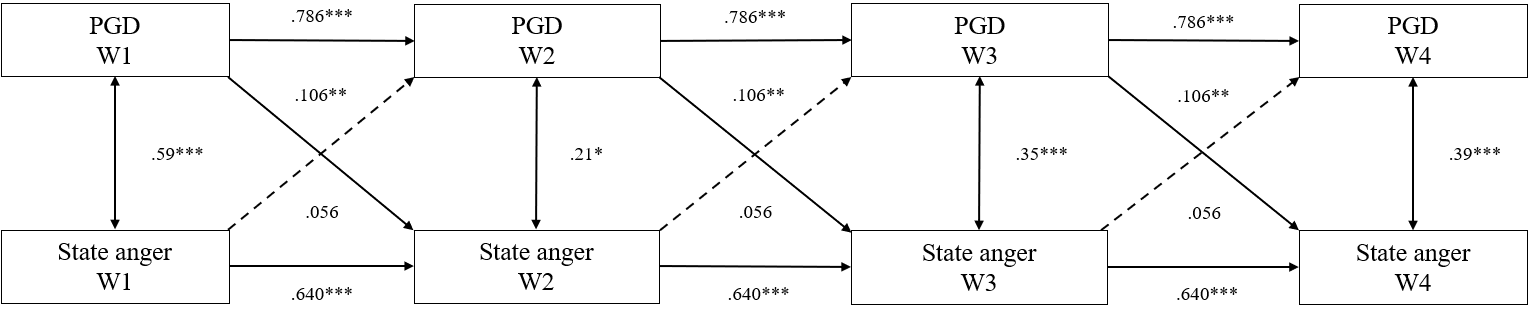
Unstandardized Estimates and Standardized Concurrent Associations Between PGD and State Anger (N* = 237)

*Note.* *** *p* < .001, ** *p* < .01, * *p <* .05. PGD = Prolonged Grief Disorder; W1 = pre-trial (67 months post-loss); W2 = pre-statement (79 months post-loss); W3 = post-statement (88 months post-loss); W4 = post-trial (103 months post-loss). Dashed lines represent non-significant paths.

**Supplementary Figure 2**

*
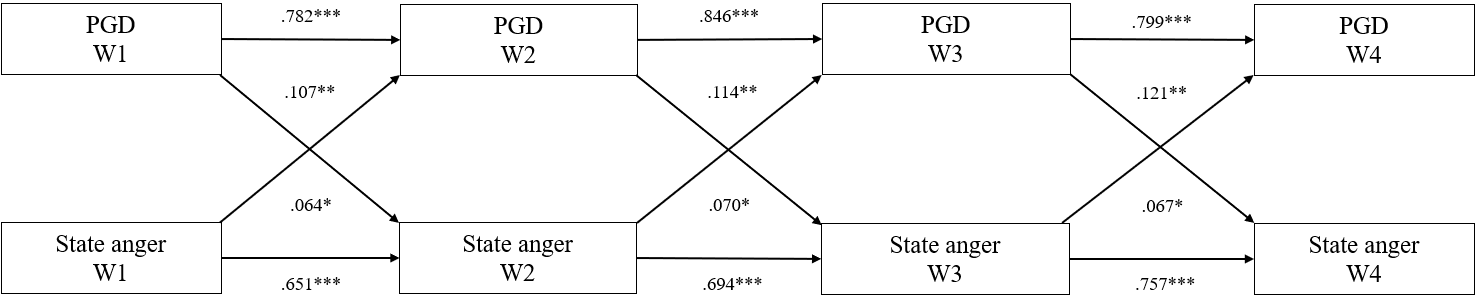
Standardized Autoregressive and Cross-lagged Paths Between PGD and State Anger with Predictors and Covariates (N* = 237)

*Note.* *** *p* < .001, ** *p* < .01, * *p <* .05. PGD = Prolonged Grief Disorder; W1 = pre-trial (67 months post-loss); W2 = pre-statement (79 months post-loss); W3 = post-statement (88 months post-loss); W4 = post-trial (103 months post-loss). The concurrent associations between PGD and state anger at each wave are not shown.

**Supplementary Figure 3**

*
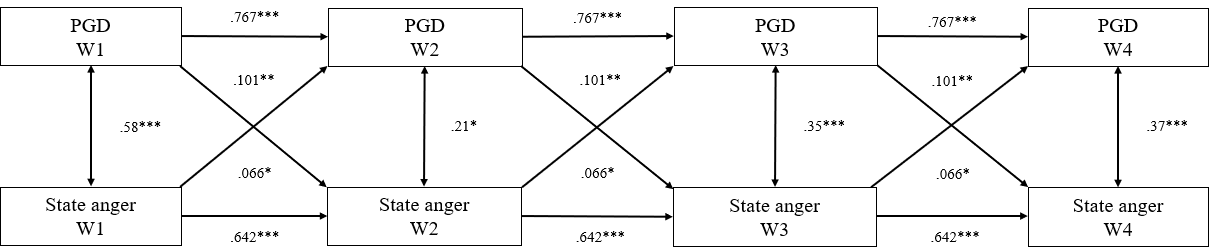
Unstandardized Estimates and Standardized Concurrent Associations Between PGD and State Anger with Predictors and Covariates (N* = 237)

*Note.* *** *p* < .001, ** *p* < .01, * *p <* .05. PGD = Prolonged Grief Disorder; W1 = pre-trial (67 months post-loss); W2 = pre-statement (79 months post-loss); W3 = post-statement (88 months post-loss); W4 = post-trial (103 months post-loss).
